# Some of the Causes of the Decay of the Teeth of Americans

**Published:** 1878-06

**Authors:** 


### ARTICLE III.

### *Some of the Causes of the Decay of the Teeth of Americans.*

BY PROF. HODGKIN.

Read before the Medical and Surgical Society of Baltimore, April 4th, 1878.

Mr. President and Gentlemen.—As physicians, you are well aware of the fact that a very large class of the ills and ails of infants that fall under your professional care, are caused, directly or indirectly, by difficult or defective or retarded dentition; and the medical books are full and medical practice is loaded with works of this class. It is not my province to discuss this subject of dentition, or, as our English friends call it, *teething*, but only in passing to call your attention to the fact that these temporary teeth are in this day oftener lost by caries than by the normal and physiological process of shedding, and to ask your attention also to a fact which is peculiar to these teeth—*i. e.*, the position of decay in the lower molars. We find that the temporary teeth are attacked by caries on their grinding and proximate surfaces, much the same as in the adult; but there is, in addition, a peculiar point of decay, so seldom met with in the adult as to exclude it from classification, and that is the location of the affection on the lingual surfaces of the lower temporary molars. Now, caries is found almost everywhere else than at this point in adult teeth, and it is worth while to bear this fact in mind, as it may be of service to us in some future discussion; and possibly some of you may be able to throw some light upon it to-night.

Normally, the temporary teeth are displaced by absorption of their roots, and the eruption of the permanent teeth follows in due course of time, and in regular order. This is *normal* teething. But in our day, such is the condition into which our children have fallen, that we find the temporary teeth are lost by caries rather than by shedding, and that the eruption of those which are expected to be permanent (though the term is almost a misnomer) takes place in edentulous jaws, the predecessors of these having been extracted to relieve the little sufferer of agonizing pain.

Let me call your attention to some peculiarities in the process of second dentition. You are aware that the first set consists of ten teeth for either jaw. You are also, of course, familiar with the beautiful physiological processes by which these teeth are evolved—beginning almost with conception and ending in the twentieth year. The details I will not burden you with—only asking your special attention to the fact that the first permanent molar is anatomically a member of the second or permanent set, but physiologically it belongs with the deciduous teeth. For while this tooth is erupted *with* the permanent set, it is formed in a sac not evolved from the temporary, as are the other permanent teeth, but is an occupant of the primitive dental groove.

It is so well known by dentists that this tooth—the first permanent molar—is the first usually to decay, that, were I addressing dentists, I should feel called on to apologize for mentioning so trite a subject. But, while as specialists we are expected, and ought to be, perfectly conversant with such facts, you who are physicians cannot be called to so strict an account. It is a fact, then, gentlemen, that the first permanent or six-year molar, is, of all the teeth, the most susceptible to decay, and is most frequently lost.

I pass over the eruption of the other teeth until we come to the canine, which is erupted about the twelfth year, and followed closely by the second permanent molars; and the case is concluded by the third molar or wisdom tooth, which appears about the twentieth year.

So much for the order of eruption, and it is important to bear this in mind, that we may fairly understand the case before us; for it is a strange fact that nearly in the order of their eruption are these teeth affected by caries. I have no time, nor can I think of burdening you with the details of the relative liability of these teeth to decay; but I simply wish to fix in your minds the fact that the first permanent molars are the first to go; the order of loss is nearly regular —*i. e.*, the next erupted are the next to fall, and so on. It may be objected that the wisdom tooth is often, very often, lost, which I admit, but the causes influencing its loss are such as do not affect the statement; for, while it is dense in structure, it is the most difficult of all the teeth to keep clean.

Now, I have stated my case. I have stated what are most generally known facts among dentists—that in the order in which teeth are evolved and erupted they are lost by decay. And you will understand that in the few minutes I have to speak to you, I cannot go into minute particulars, or burden you with what few of us delight in—statistics.

Why is it that we lose our teeth in this way? Why is it that these organs which nature certainly intended should serve the purpose of mastication throughout our three-score years and ten, and be buried with us at last—why is it that they lie scattered along the pathway of our lives, ornamenting the collection of curiosities of some dentist; or, as irritating stumps, be the cause of endless annoyance and pain? While we are yet in our youth we are perforce (and this in spite of the vast progress which is being made in the art of preserving the teeth by filling them)—in spite of the fact that our knowledge of hygiene in other directions is prolonging life, and rendering it more tolerable to the feeble—in spite of all this, many are wearing artificial substitutes for natural organs, and the proportion of such is annually increasing, until the very children are losing these priceless treasures.

Some deep-seated cause is here. The immediate cause of dental caries we infer to be an acid condition of the secretions of the oral cavity, added to the effect produced by the decomposition of particles of food retained between the teeth. But behind all this is the plain fact, staring us in the face, that this is only the *proximate* cause, and that the predisposing cause must lie deeper. This is plain from the fact that the teeth of the lower omniverous domesticated animals—the dog, cat, &c., used in the mastication of substantially the same food, are not attacked by decay—indeed, in these animals, are rarely lost at all.

The loss of teeth by caries is peculiar to the human race, and peculiar only to the more civilized part of that race, and peculiarly to the Anglo-American. I am not prepared to say to what extent Europeans lose their teeth, but the impression prevails that they do not do so to the extent that we do; and as for the semi-savage and barbarous tribes, they are exempt from this curse in the main.

I have referred to the order in which the teeth are erupted—and have indicated that it is in the order of their development. We have seen further that these processes date far back in the history of the yet unborn child. In that mysterious evolution of which we know so little—in that occult process of the "growth of the bones in the womb of her that is with child," so very early in the life of the fœtus as to excite incredulity when the statement is made to the unscientific—the germs of the deciduous and the permanent teeth are formed; and before birth, their character is more or less firmly fixed for good or for ill.

In this wonderful laboratory of nature, where pulp and dentine, enamel and cementum are formed, where calcification is progressing, and "form and substance" are being given to organs which are as yet in embryo, a slight cause may disturb the balance of forces and effect the nutrition of these teeth.

A nervous, over-worked man, with feeble muscles and imperfect digestion, with brain force up to its highest tension, dyspeptic and prematurely old, comes from a day of harrassing care, at night to share the bed of a hysterical, flabby-muscled anæmic woman, all of whose organs are out of tone, and whose whole life-force is exhausted in her monthly periods. Too little of a woman to be other than passive under his conjugal embraces, too feeble for anything except the unfortunate inevitable conception which takes place, altogether unfit for the conditions which maternity imposes—this makeshift of a woman—this absorption of a true mother—carries in her for nine months the babe which she, at the fullness of time, brings all immature and imperfectly developed into the world. The history of those nine months is better known to you than to me. The utter want of tone, the absence of all elasticity, the yielding up to languor and debility, the defective nutrition—too often is it the case that the maternity is undesired, and the mournful spectacle is seen of an unwilling mother. Can there be a more lamentable condition of affairs? Can there be any hope, except that "great nature is more wise than we?"

Is it any wonder that the development of this child is bad? Is it any matter of mystery that we find it like its parents? Its after-history is seen in the record of your vital statistics, which show that half of these unfortunates perish by the age of five years, and that half of the survivors die of consumption.

Some survive, thanks to the admirable hygiene of our day. Good nursing, skillful medication, all the arts of modern science are brought into requisition, and the lines of some are prolonged until they outgrow their feebleness. Our physiology teaches us that their bones change, their tissues moult and are cast off, that their structures, by exercise and the beneficent help of a Providence, which *tends* to set us upright, no matter how we may be warped by malformation, are improved, until the feeble child of feeble parents is developed into something of robustness—only *something*, for the constitution is, as we know too sadly, anything but good, and succumb readily to disease.

But the same physiology teaches that while these improvements are being made in the tissues—the bones, muscles, &c.—the *teeth* are not susceptible to such change. They are *calcified*, not ossified, and as they are formed so they remain. The truth *seems* to be that the enamel, the outer covering of the tooth—hard, dense, almost vitreous and practically inorganic—when once formed, can undergo no change of a physiological character, but remains throughout life as when first calcified.

Now, when we revert to the statements made earlier in this paper—to the fact that those teeth which are first formed are first to be destroyed by caries ; that the temporary teeth are often attacked the first year of this eruption ; that the first permanent molars, which belong there physiologically, are almost as easily destroyed ; that the canines and wisdom teeth, which are matured in extra-uterine life, and when the tissues have opportunity for more perfect nutrition, are the strongest of all—I come to the conclusion that the great predisposing cause of the decay of the teeth of Americans is that of defective intra-uterine development.

Of course it is not for me to suggest (nor do I understand that this is the place or time) the remedy. And we all know perfectly well that argument would be useless in the presence of the mightier and all-potent, irresistible influence which impels marriages of unphysiological character. The feeble will mate with the frail I fear to the end of time, and what has been I suppose will be. But we can, perhaps, help by a few suggestions the condition of matters, which is certainly lamentable enough. You, gentlemen, of the medical profession, are in a better position than we of the dental profession to advise and counsel those who are bringing into the world so many delicate children, with imperfectly developed organs and structures, and you can earnestly advise the use of such hygienic means as may tend to modify this condition of affairs. Get your patients to understand that the function of gestation is of prime importance, not simply an accident of coition ; that maturity is sacred, that the chances of having healthy offsprings are greatly increased by the approximate perfection of the physical conditon, and that mothers may at least tend to secure better teeth for their children by bringing about in themselves the highest type of development. All that can influence the love of a mother for her child, its future welfare, its strength, its constitution, she should know; and should know also that her mental condition is likely to affect her child's future.

I sum up, then, my conclusions (and I will be glad to have any light upon a subject about which I feel that I know less even than most of my hearers:)

1. That as teeth formed in structure, so they remain.

2. That they decay in somewhat the order, all other things being equal, of their calcification and eruption.

3. That teeth which are calcified in extra-uterinel ife, if the conditions of calcification are favorable, are less liable to decay.

4. That the condition of the mother, and possibly of both parents, materially influences the condition of the teeth so far as their calcification is concerned, and consequently their durability.

5. That the teeth of animals living more physiologically than civilized man, are not subject to dental caries; and that: Finally, good teeth will withstand even ill treatment. —*Virginia Med. Monthly.*

---

